# Identification and functional activity of Nik related kinase (NRK) in benign hyperplastic prostate

**DOI:** 10.1186/s12967-024-05048-3

**Published:** 2024-03-09

**Authors:** Weixiang He, Zelin Tian, Bingchen Dong, Yitong Cao, Wei Hu, Peng Wu, Lei Yu, Xinhua Zhang, Shanshan Guo

**Affiliations:** 1 Department of Urology, Xijing Hospital of Air Force Medical University, West Changle Road 127, Xi’an, China; 2 Department of Hepatobiliary Surgery, Xijing Hospital of Air Force Medical University, Xi’an, China; 3 Department of Orthopedics, Ninth Hospital of Xi’an, Xi’an, China; 4 Department of Urology, Zhongnan Hospital of Wuhan University, Donghu Road 169, Wuhan, China; 5 Department of Physiology and Pathophysiology, Air Force Medical University, West Changle Road 169, Xi’an, China

**Keywords:** Benign prostatic hyperplasia, Nik-related kinase, Lower urinary tract symptoms, Bioinformatics, Fibrosis, EMT

## Abstract

**Objective:**

Benign prostatic hyperplasia (BPH) is common in elder men. The current study aims to identify differentially expressed genes (DEGs) in hyperplastic prostate and to explore the role of Nik related kinase (NRK) in BPH.

**Methods:**

Four datasets including three bulk and one single cell RNA-seq (scRNA-seq) were obtained to perform integrated bioinformatics. Cell clusters and specific metabolism pathways were analyzed. The localization, expression and functional activity of NRK was investigated via RT-PCR, western-blot, immunohistochemical staining, flow cytometry, wound healing assay, transwell assay and CCK-8 assay.

**Results:**

A total of 17 DEGs were identified by merging three bulk RNA-seq datasets. The findings of integrated single-cell analysis showed that NRK remarkably upregulated in fibroblasts and SM cells of hyperplasia prostate. Meanwhile, NRK was upregulated in BPH samples and localized almost in stroma. The expression level of NRK was significantly correlated with IPSS and Q_max_ of BPH patients. Silencing of NRK inhibited stromal cell proliferation, migration, fibrosis and EMT process, promoted apoptosis and induced cell cycle arrest, while overexpression of NRK in prostate epithelial cells showed opposite results. Meanwhile, induced fibrosis and EMT process were rescued by knockdown of NRK. Furthermore, expression level of NRK was positively correlated with that of α-SMA, collagen-I and N-cadherin, negatively correlated with that of E-cadherin.

**Conclusion:**

Our novel data identified NRK was upregulated in hyperplastic prostate and associated with prostatic stromal cell proliferation, apoptosis, cell cycle, migration, fibrosis and EMT process. NRK may play important roles in the development of BPH and may be a promising therapeutic target for BPH/LUTS.

**Supplementary Information:**

The online version contains supplementary material available at 10.1186/s12967-024-05048-3.

## Introduction

Benign prostatic hyperplasia (BPH) and its related lower urinary tract symptoms (LUTS) are very common in elder men, which affecting their quality of life. Despite current medical therapy such as α1-adrenoceptor antagonists (α1-blockers) and 5α-reductase inhibitors (5-ARIs) serve satisfactory, a portion of patients might experience complications or require further surgical interventions [1]. The dilemma in medical therapy of BPH/LUTS was probably due to its not-fully revealed etiology and pathogenesis. Further and in-depth of investigations for therapeutic targets are of great significances. To date, aging and testosterone are two defined independently risk factor of BPH/LUTS. With regard to the pathogenesis of BPH/LUTS, some hypothesis have been proposed including growth factor, stromal-epithelial interaction, ratio between estrogen and androgen, chronic inflammation, genetic and familial factors, metabolic syndrome, autoimmunity and embryonic reawakening theory [1–3]. However, from the pathological aspect, besides the continuous nonmalignant proliferation of prostate cells, BPH tissues also exhibit stromal fibrosis [4–6]. Stroma myofibroblasts play pivotal role in the process of fibrosis that accumulation of which results in remodeling of the extracellular matrix (ECM) and tissue hardening by secreting large amounts of collagen proteins deposited [7]. Fibrosis was proposed to be a new pathological mechanism of BPH and involved in the progress of LUTS [6, 8, 9], however, the current first-line oral medications (α1-blockers and 5-ARIs) have shown no effect on reverse or alleviation of fibrosis. Meanwhile, epithelial-mesenchymal transition (EMT) also contributes to prostatic fibrosis and hyperplasia, which leads to BPH/LUTS progress [4, 10, 11]. EMT is characterized as increasing mesenchymal-like cells derived from the prostatic epithelium and TGF-β is thought to play a key role in EMT [4, 12]. Therefore, fibrosis and EMT might be a promising therapeutic target for BPH/LUTS.

With the development of bioinformatics analysis in recent years, many studies have given new insight into pathophysiology mechanism of BPH. Functional activity and related pathways of biomarkers including BMP5, NELL2, IGF1, CXCL13, MXRA5 and GPX3 were identified from many analyses using gene microarrays and RNA-seq datasets [13–19]. A recent study also investigated therapeutic targets in BPH using single-cell RNA-seq (scRNA-seq) data [20]. However, integrative analysis using multiple datasets are few. Our current analysis combined RNA-seq and scRNA-seq datasets to explore the possible biomarkers of BPH and demonstrated that the Nik related kinase (NRK) might be a new therapeutic target for BPH/LUTS.

NRK, a gene on the X-chromosome and a member of the germinal center kinase (GCK) family subgroup IV, was firstly cloned from mice and found in skeletal muscle during mouse embryogenesis in 1996 [21]. Most studies of NRK focused on its crucial role of regulating placenta development [22–26]. In other organs or tissues, NRK also exhibit different functions including modulating cell proliferation [27], apoptosis [28, 29], migration [30], cell cycle [31], inflammation regulation [32] and cytoskeletal organization [33]. However, the expression of NRK in human prostate and its role in development and progress of prostatic disease have never been elucidated.

Therefore, current study would explore the new biomarkers of BPH using integrative bioinformatics analysis and investigate the expression and the functional activity of NRK in hyperplastic prostate.

## Methods

### Bulk RNA microarrays obtained and DEGs identification

Raw gene expression profiles (GSE7307, GSE119195 and GSE132714) were obtained from Gene Expression Omnibus (GEO) database (http://www.ncbi.nlm.nih.gov/geo/), the related information was listed in Table 1. These three datasets were combined and analyzed to screen differentially expressed genes (DEGs). As described in our previous studies [18], R software (version 4.3.1) and related packages were used to identify the DEGs of each dataset. The cut-off standards for DEGs were p < 0.05 and |logFC|> 1. The gene expression level of each dataset was presented in individual volcano chart using Sangerbox (http://vip.sangerbox.com/home.html). In addition, the Venn diagram tool from Sangerbox was applied to overlap the DEGs from above three datasets, which were then labelled in volcano chart, respectively.

**Table 1:** Clinical information of BPH patients

Items	
No. of patients	104
Age (year)	70.1
BMI (kg/m^2^)	22.8
Prostatic size (ml)	60.8
tPSA (ng/ml)	7.0
fPSA (ng/ml)	1.6
IPSS	21.7
Q_max_ (m/s)	10.0
PVR (ml)	160.7

### GO and KEGG pathway enrichment analysis

Above identified DEGs were submitted to DAVID website (https://david-d.ncifcrf.gov/). GO (Gene Ontology) and KEGG (Kyoto Encyclopedia of Genes and Genomes) pathway outcome were analyzed and cutoff values were p < 0.01. R package DOSE was used to analysis the relationship between genes and pathways.

### Single cell RNA-seq analysis

Similar to bulk RNA microarray, raw data of single cell dataset (GSE172357) were also downloaded from GEO database. The detailed information of GSE172357 were also listed in Table 1 and R software (version 4.3.1) was used for further analysis. Packages of R software including NormalizeData, FindVariableFeatures, and ScaleData were applied to normalize the scRNA-Seq data. Then, dimensionality of the data were reduced the to a two-dimensional (2D) space by package Seurat() and then visualized by T-distributed stochastic neighbor embedding (tSNE). FindNeighbors, FindClusters, and RunTSNE were used to perform these analyses with a resolution of 0.1–0.5. According to the canonical patterns of marker genes, the 29,071 cells were annotated as 15 cell types including macrophages, fibroblasts, acinar cells, endothelials, monocytes, neutrophils, progenitor cells, ductal cells, dendritic cells (DC-cell), stellate cells, α-cells, β-cells, T cell, B cell, and granulocytes. The main reference databases of cell marker genes for cell annotation came from CellMarker (http://xteam.xbio.top/CellMarker/index.jsp, accessed on 1 September 2022) and CellMarker2.0 (http://bio-bigdata.hrbmu.edu.cn/CellMarker/index.html, accessed on 1 September 2022).

### Cell clusters-specific metabolic pathway analysis

To investigate cell type-specific metabolic pathway features, we sought to quantify pathway activity and describe it as a score [34]. According to this score, it could determine whether metabolic pathway activity is related to specific cell type, and identify metabolic pathways that were specifically activated by one cell type. The value *p*_*t,j*_ represented the activity of the *t* pathway in the *j* cell type, which calculation formula is as follow:

$$ p_{t,j} = \frac{{\sum\nolimits_{i = 1}^{{m_{t} }} {\omega_{i} \times r{}_{i,j}} }}{{\sum\nolimits_{i = 1}^{{m_{t} }} {\omega_{i} } }} $$

The value *m*_*t*_ is the number of genes in the *t* pathway. The value *ω*_*i*_ is the weighting factor representing the reciprocal of the number of pathways containing the *i* gene. The value *r*_*i,j*_ is the relative quantification of the average expression level of the *i* metabolic gene in *j* cell type versus all types of cells. If *r*_*i,j*_ > 1, it is proved that the average expression value of the *i* gene in the *j* cell type is higher than that in all types of cells. The calculation formula of *r*_*i,j*_ is as follow:

$$ r_{i,j} = \frac{{E_{i,j} }}{{\frac{1}{N}\sum\nolimits_{j}^{N} {E_{i,j} } }} $$

The value *N* is the total number of cell types. The value *E*_*i,j*_ is the average expression level of the *i* metabolic gene in the *j* cell type, which calculation formula is as follow. The value *n*_*j*_ is the number of cells in the *j* cell type. The value *g*_*i,k*_ is the expression level of the *i* gene in the *k* cell. The value *M* is the total number of all metabolic genes.

$$ {\text{E}}_{i,j} = \frac{{\sum\nolimits_{k = 1}^{{n_{j} }} {g_{i,k} } }}{{n_{j} }},\quad i \in 1 \ldots M,j \in 1 \ldots N $$

### Human prostate tissues

Human prostate tissues were obtained from 3 young brain-dead men who underwent organ donation in Xijing Hospital of Air Force Medical University as controls (from May 2022 to June 2022). Informed Consent Form about organ donation were signed before donation and these three organ donors or their next of kin agreed to donation and were aware of the organ use. The prostate samples of 104 BPH patients with clinical data who underwent transurethral resection of the prostate in the Department of Urology, Zhongnan Hospital of Wuhan University were collected (from March 2018 to January 2019). The sample size was determined based on further pathological confirmation. Postoperative pathological examination confirmed the diagnosis of BPH. Prostate tissues were divided into two parts, which were stored in liquid nitrogen for PCR and Western blot analysis and in 4% PFA for histological detection. All human specimens were collected and processed in accordance with the guidelines approved by the Ethics Committee and the principles of the Declaration of Helsinki.

### Tissue microarray (TMA) construction and Immunohistochemistry

Detailed information of the clinical characters of 104 BPH patients are presented in Table 1. Tissue from each of the 104 patient cases was fixed, made into donor wax block sections. A 1.5 mm diameter core of was taken from each sample wax block to construct TMA, and then serially cut into sections with a thickness of 4 μm for further staining.

The paraffin sections were deparaffinized in xylene prior to anhydrous ethanol, 95% alcohol and 75% alcohol in turn. They were subsequently kept in 10 mM boiled sodium citrate buffer (pH 6.0) for 2 min for antigen retrieval and incubated with 3% H_2_O_2_ solution for 10 min to inactivate endogenous peroxidase. To block non-specific binding, 15% normal goat serum was used to incubate sections for 15 min at room temperature. Next, the sections were incubated successively with primary antibodies (Additional file 1: Table S1) at humidified and 4 °C conditions and with secondary antibody at 37 °C until peroxidase and 3, 3′-diaminobenzidine tetrahydrochloride visualization. Negative controls were incubated with PBS instead of the antibody. All stained sections were imaged using an Olympus-DP72 light microscope (Olympus, Japan).

### Correlation analysis

Expression of NRK, E-cad, N-cad, α-SMA and Collagen-I in the prostate tissues from our TMA were blindly quantified by two pathologists via analysis for positive area of all images with Image J. Pearson correlation analysis was conducted to investigate the correlation between NRK and related proteins (E-cad, N-cad, α-SMA and Collagen-I). Meanwhile, and correlation between several clinical characteristics of BPH and NRK expression level was also analyzed.

### Human prostatic cell lines

Two prostatic cell lines BPH-1 and WPMY-1 were used for our current study. BPH-1, human benign prostatic enlargement epithelia cell line (Cat. #BNCC339850, purchased from the Procell Co., Ltd., Wuhan, China), was cultured in RPMI-1640 medium (Gibco, China) containing 10% fetal bovine serum (FBS) (Gibco, Australia). WPMY-1, SV40 large T antigen-immortalized stromal cell line (Cat. #GNHu36, purchased from the Stem Cell Bank, Chinese Academy of Sciences, Shanghai, China), was cultured in DMEM medium (Gibco, China) containing 1% penicillin G sodium/streptomycin sulfate and 5% FBS. All cells were cultured in a humidified atmosphere consisting of 95% air and 5% CO_2_ at 37 °C. Identification of the cell lines was performed at the China Center for Type Culture Collection in Wuhan, China.

### Knockdown of NRK in prostatic cells

NRK-target specific small interfering RNAs (siRNAs) were synthesized by Genepharma Ltd. in Suzhou, China. WPMY-1 cell lines were transfected with NRK-siRNAs using lipofectamine 2000 (Invitrogen, USA), according to the manufacturer’s instruction. The sequences of si-NRKs are listed in Additional file 2: Table S2. After transfection by si-NRK for 48 h, alterations of NRK at transcriptional and protein levels were evaluated by the qRT-PCR, Western-blot. The efficacy of knockdown were screened in Additional file 4: Figure S1 and si-NRK-1 and si-NRK-2 were used for further study. The si-NRK-1 was used for rescue experiment.

### Plasmid construction and cell transfections

NRK plasmid used in this study were obtained from Hanbio Biotechnology Co. Ltd (Shanghai, China). DNA sequencing technology was performed to verifying the sequence of all plasmids. Plasmid transfection was performed using Lipofectamine 2000 reagent (Invitrogen) according to the manufacturer's instructions. The efficacy of overexpression was screened in Additional file 4: Figure S2.

### Drug treatment

In order to induced EMT process of prostate epithelial cells [35], BPH-1 cells were pretreated with 10 μg/L TGF-β1 (MedChemExpress, China) for 24 h prior to siRNA transfection. In addition, in order to induced prostate stromal cells fibrosis, WPMY-1 cells were pretreated with 0.27238 μg/l (equivalent to 1 nM) estradiol (E_2_, MedChemExpress, China) for 24 h prior to siRNA transfection [36]. Meanwhile, equivalent amount of DMSO without drugs were added to the cells served as a control.

### Cell proliferation assay

WPMY-1 cells were seeded in 6-well plates and transfected with siNRK-1, or siControl. After 24 h, cells were washed and counted. Cell Counting Kit-8(CCK8) (C0005, TargetMol), 5-ethynyl-20-deoxyuridine (EdU) (C10310-1, RiBoBio) were used to assess cell proliferation following the manufacturer’s protocol.

### Flow cytometry analysis for cell cycle and apoptosis

1 × 10^6^ cells were harvested for cell cycle and apoptosis analyses. For cell cycle analysis, cells were centrifuged and then pellets were resuspended with PBS containing 50 μg/ml propidium iodide (Multi Sciences, Hangzhou, China) and 0.1 mg/ml RNaseA (20 μg/ml in PBS) in the dark. After incubation at 37 °C for 30 min, the DNA content distribution was analyzed by flow cytometry analysis (Beckman, Cat. #FC500). Cell apoptosis was analyzed by flow cytometry using the Annexin V-FITC apoptosis analysis kit (Sungene Biotech., Tianjin, China), according to the manufacturer’s instructions.

### Wound-healing assay

Prostatic cells were seeded into 6-well plates and cultured until full confluent, then the cell monolayer was scratched in a straight line using a 200 μl sterile pipette tip and washed with PBS three times. Cells were maintained in serum-free medium and images were captured every 24 h.

### Transwell migration assay

Migration capability was measured using 24-well transwell chamber systems (Corning, USA) with 8.0 µm pore size. Cells were seeded in the upper chamber insert and cultured in serum-free medium. The bottom chambers were filled with 10% fetal bovine serum medium. After 24 h, the migrated cells were fixed with 4% paraformaldehyde for 20 min at room temperature and then stained with 1% crystal violet. The migrated cells were counted and photographed in three randomly selected views.

### Total RNA extraction and cDNA synthesis

As described in a previous study from our group [37], total RNA was isolated from tissues and cells using TRIzol reagent (Invitrogen, Carlsbad, CA, USA) according to the manufacturer’s protocol. One μg of total RNA was reverse-transcribed to cDNA via the SuperScript II First-Strand Synthesis System according to the instructions (Invitrogen).

### Real-time reverse transcriptase polymerase chain reaction (real-time RT-PCR)

As previously described [18], RT products were amplified in a 96-well plate in a 20 μL reaction volume with all samples run in triplicate, using the CFX Connect Real-Time PCR System™ (Bio-Rad). The following experimental protocol was utilized: denaturation (95 °C for 10 min to activate the polymerase) followed by an amplification program repeated for 40 cycles (95 °C for 15 s, then 60 °C for 60 s) using a single fluorescence measurement. Primer sequences are shown in Additional file 3: Table S3. Target genes were amplified using SYBR Green for amplicon detection. For relative quantification, gene expression was normalized to expression of the GAPDH ribosomal housekeeping gene as an internal control and compared by 2^−ΔΔCT^ method.

### SDS-PAGE and western-blotting analysis

As previously described [18], proteins were extracted from tissues and cells using RIPA (Radio-Immunoprecipitation Assay) lysis buffer (Sigma-Aldrich, St Louis, Mo) (containing 1 × PBS, 1% IGEPAL CA-630, 0.5% sodium deoxycholate, 0.1% SDS, 10 mM EDTA) with freshly added phenylmethanesulphonylfluoride (PMSF; Sigma Aldrich, St. Louis, MO, USA) and sodium orthovanadate (Sigma Aldrich). An aliquot of 100 μg of each sample was electrophoresed on a 7.5% or 12.5% sodium dodecyl sulfate–polyacrylamide (SDS-PAGE) gel (Wuhan Boster Biological Technology Ltd, Wuhan, China) and transferred to polyvinylidene fluoride (PVDF) membrane (Millipore, Billerica, MA, USA) using a Bio-Rad wet transfer system. The membrane was blocked for 2 h at room temperature with Tris-buffered saline with 0.1% [v/v] TBST containing 5% [w/v] non-fat dry milk. The membranes were incubated overnight with corresponding protein primary antibodies (Information of primary antibodies was listed in Additional file 1: Table S1). After washing several times, the membranes were incubated with secondary antibody at room temperature for 2 h. Detection of reaction antigen was performed with an enhanced chemiluminescence (ECL) kit (Thermo Scientific Fisher, Waltham, MA, USA). The bands were quantified by reflectance scanning of gel photographs obtained with a BioDoc XRS + camera using Bio-Rad Molecular Imager® ChemiDoc™ XRS + System and Quantity One® SW 1-D Analysis Software (Bio-Rad).

### Statistical analysis

Results are expressed as mean ± SD of at least three independent experiments. Statistical analysis was performed using Excel software. For two groups, Student’s t-test was used to determine statistical significance. One-way analysis of variance (ANOVA) was used for multiple group comparisons. The statistical test methods used in bioinformatic analysis were according to related R package in each section. *p* < 0.05 was considered significant. Asterisks represent the level of significance: *p < 0.05, **p < 0.01, ***p < 0.001, ****p < 0.0001. P-values in current study are descriptive and as measures of central tendency.

## Results

### Bulk RNA-seq analysis reveals DEGs between BPH and control groups

**Fig. 1:**
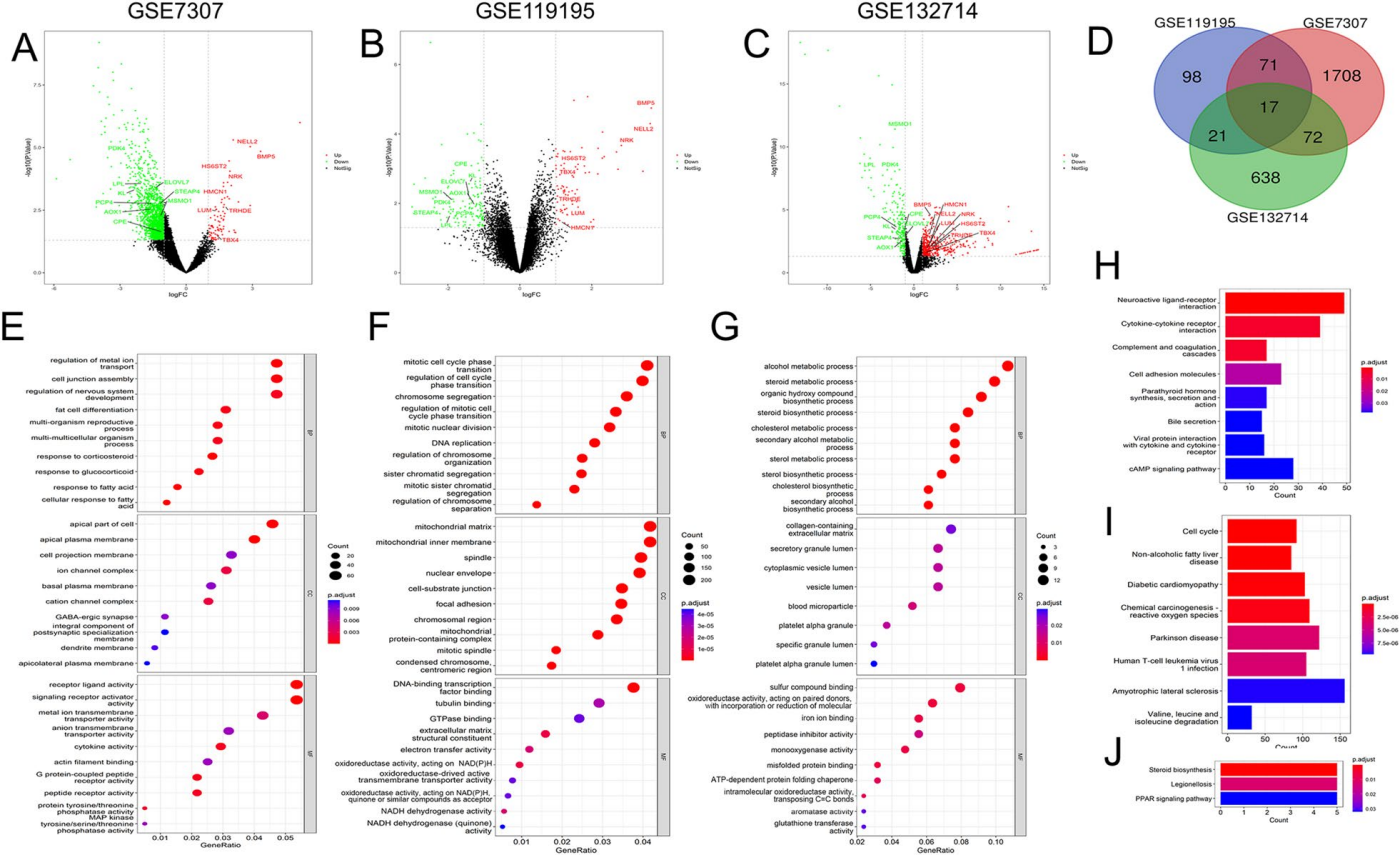
DEGs in BPH and results of GO, KEGG enrichment analyses. **A**–**C** Volcano plot of DEGs in GSE3707, GSE119195, GSE132714; **D** Venn diagrams showed the number of DEGs that overlap between GSE3707, GSE119195, GSE132714; **E** The enriched GO terms of DEGs in GSE3707; **F**The enriched GO terms of DEGs in GSE119195; **G** The enriched GO terms of DEGs in GSE132714; **H** KEGG pathway enrichment results in GSE3707; **I** KEGG pathway enrichment results in GSE119195; **J** KEGG pathway enrichment results in GSE132714

Three bulk RNA-seq datasets from GEO database were obtained and analyzed for further investigation, which specific information were listed in Table 1. As shown in volcano chart, 1868 DEGs were identified from the analysis of dataset GSE7307, among which 84 were upregulated and 1784 were downregulated (Fig. 1A). 207 DEGs were identified from the analysis of dataset GSE119195, among which 77 were upregulated and 130 were downregulated (Fig. 1B). 748 DEGs were identified from the analysis of dataset GSE132714, among which 527 were upregulated and 221 were downregulated (Fig. 1C). After overlapped, 17 DEGs were identified among three datasets (Fig. 1D). Eight genes were upregulated including *BMP5, NELL2, NRK, HS6ST2, HMCN1, LUM, TRHDE* and *TBX4*, meanwhile, nine genes were downregulated including *PDK4, LPL, KL, PCP4, AOX1, CPE, ELOVL7, STEAP4* and *MSMO1*. The DEGs were further processed for functional enrichment with GO and KEGG pathway analyses. The most enriched GO terms were classified to Biological Process (BP), Cellular Component (CC) and Molecular Function (MF), majoring including regulation of nervous system development, receptor ligand activity, mitotic cell cycle phase transition, chromosome segregation, mitotic cell cycle phase transition, etc. (Fig. 1E–G). The most enriched KEGG pathways of the DEGs were dominated by pathways involved in Neuroactive ligand-receptor interaction, Amyotrophic lateral sclerosis, steriod biosynthesis, etc. (Fig. 1H–J).

### Single cell RNA-seq analysis reveals DEGs between BPH and control groups

**Fig. 2:**
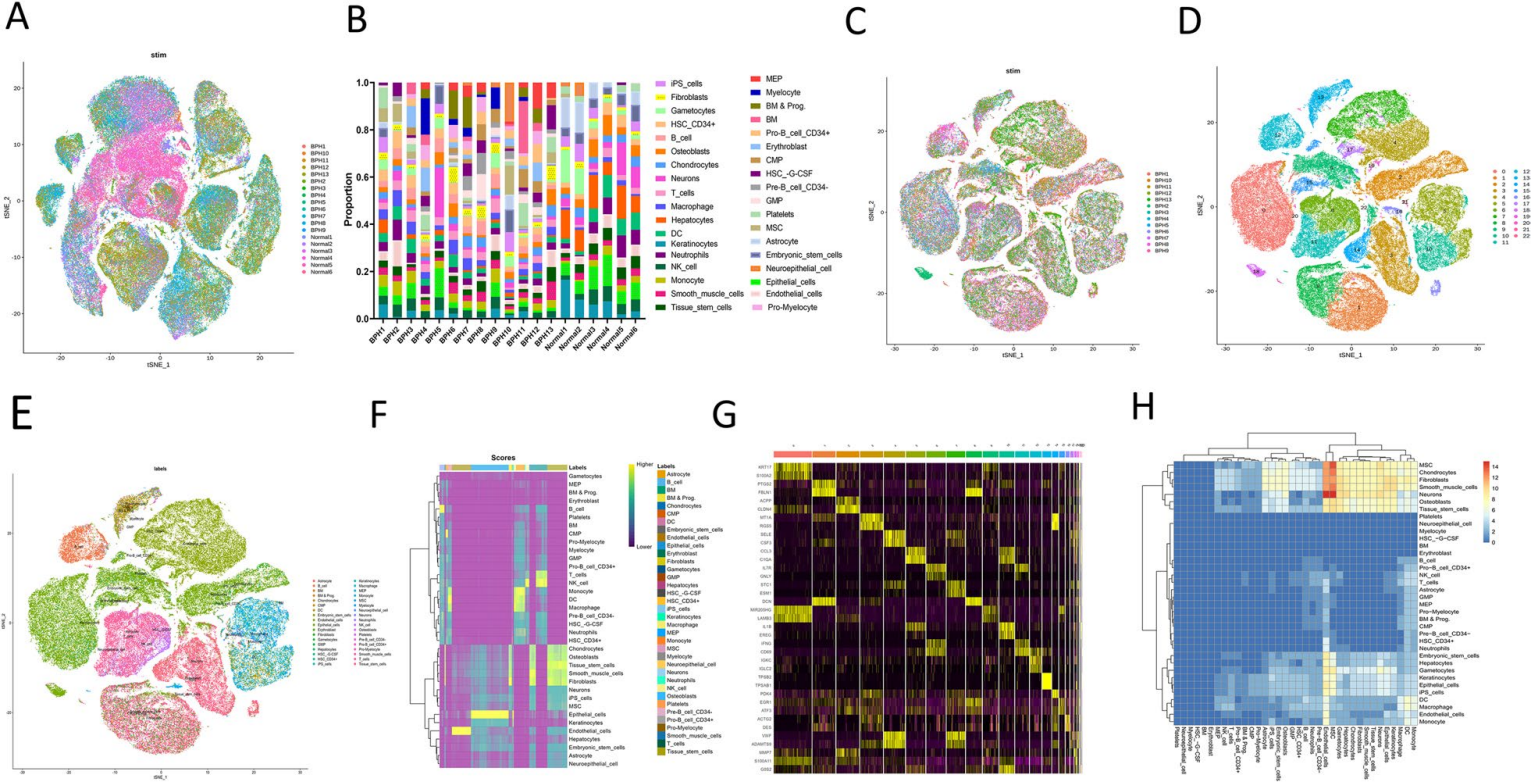
Dissection of BPH with scRNA-seq. **A** tSNE plot of 161,801 cells from 13 BPH tissues and 6 healthy control tissues; **B** The proportion of 36 cells built on scRNA-seq data. **C**–**E** tSNE plot of 115,402 cells from 13 BPH tissues (label colors are according to separate clusters and cells). **F** Cells heatmap of each cluster subpopulation. **G** Marker genes heatmap of each cell subpopulation. **H** Heatmap of all cellular communications

Data from datasets GSE172357 were obtained and analyzed for further investigation. A total of 161,801 cells were analyzed from human prostate samples, of which 46,399 were from 6 control samples and 115,402 cells were from 13 hyperplastic prostate samples, respectively (Fig. 2A). The singleR is used to annotate all cells, and the proportion of 36 cell types in each sample is shown in Fig. 2B. As shown in Fig. 2C, D, 23 major cellular clusters were identified in 115,402 hyperplastic prostate cells from 13 hyperplastic prostate samples. The marker genes of each cluster were identified and analyzed. According to the canonical patterns of these marker genes, above-mentioned 115,402 hyperplastic prostate cells were annotated as 36 cell types including macrophages, fibroblasts, acinar cells, endothelials, monocytes, neutrophils, progenitor cells, ductal cells, DC-cells, stellate cells, α-cells, β-cells, T cells, B cells, and granulocytes (Fig. 2E). The cluster heatmap of these cell types is shown in Fig. 2F. In addition, the expression level of marker genes for each cluster were shown in a heatmap (Fig. 2G). Cellchat is used to investigate intercellular communication of the BPH cells. The heatmap of all intercellular communication showed that the communication between endothelial cells and neurons was the most prominent. The second most prominent communication was between MSC and neurons, endothelial cells (Fig. 2H).

### Analysis of DEGs in epithelial cells, fibroblasts and SM cells cluster

**Fig. 3:**
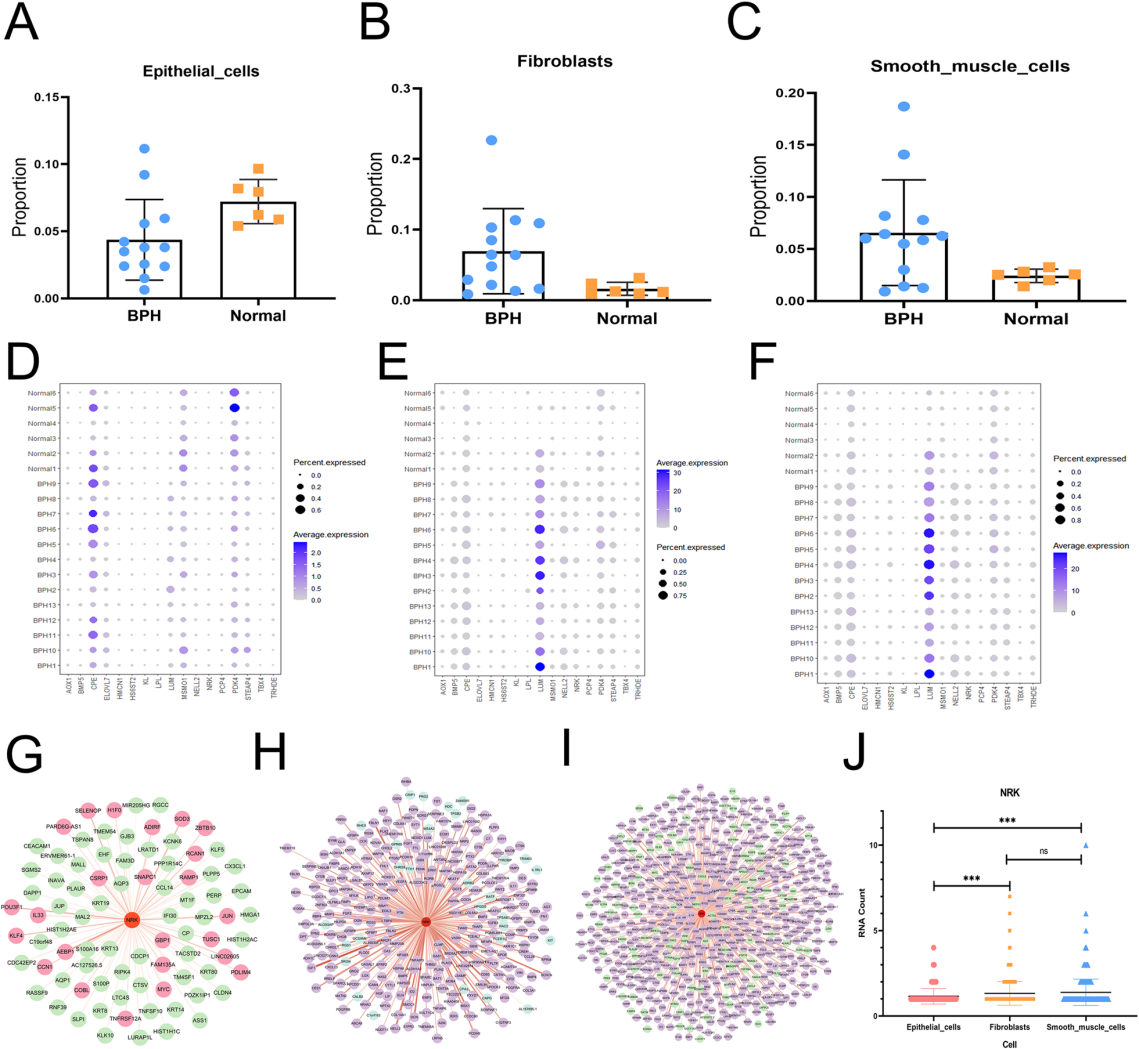
The DEGs in epithelial cells, fibroblasts and SM cells. **A** The difference of cells proportion between BPH tissues (n = 13) and healthy control tissues (n = 6) in epithelial cells; **B** the difference of cells proportion between BPH tissues (n = 13) and healthy control tissues (n = 6) in fibroblasts; **C** the difference of cells proportion between BPH tissues (n = 13) and healthy control tissues (n = 6) in SM cells; **D** the expression level of 17 DEGs in epithelial cells; **E** the expression level of 17 DEGs in fibroblasts; **F** the expression level of 17 DEGs in SM cells. **G** Gene co-expression network based on NRK and other marker genes in epithelial cells. **H** Gene co-expression network based on NRK and other marker genes in fibroblasts; **I** gene co-expression network based on NRK and other marker genes in SM cells. **J** The difference of NRK expression level between epithelial cells, fibroblasts and SM cells

We further investigation of above 17 DEGs in specific cell cluster. To our knowledge, epithelial cells, fibroblasts and SM cells are three most cell type in human prostate, therefore, these three clusters were chosen for further study. As shown in Fig. 3A–C, the proportion of epithelial cells were decreased while fibroblasts and SM cells were increased in hyperplastic prostate. Then, as shown in Fig. 3D–F, we examined the expression level of 17 DEGs in each cluster. According to 8 upregulated genes, we found *BMP5, NELL2, NRK, LUM,* and *TBX4* were also significantly upregulated in both fibroblasts and SM cells clusters from BPH patients (*p* < 0.05, respectively). Meanwhile, the expression level of *PDK4* and *MSMO1,* two of identified downregulated genes, were also decreased in epithelial cells cluster. In addition, the correlation between NRK and marker genes in epithelial cells were analyzed, from which we found 24 marker gene expressions are positively correlated with NRK, while 60 marker gene expressions are negatively correlated with NRK (Fig. 3G). Meanwhile, in fibroblasts, 252 marker gene expressions are positively correlated with NRK, while 28 marker gene expressions are negatively correlated with NRK (Fig. 3H). In SM cells, 383 marker gene expressions are positively correlated with NRK, while 60 marker gene expressions are negatively correlated with NRK (Fig. 3I). The expression level of NRK in various cell clusters were shown in Fig. 3J. However, the expression level of NRK in epithelial cells is significantly different from that of fibroblasts and smooth muscle cells (*p* < 0.001, respectively).

### Analysis of cell type-specific metabolic pathway

**Fig. 4:**
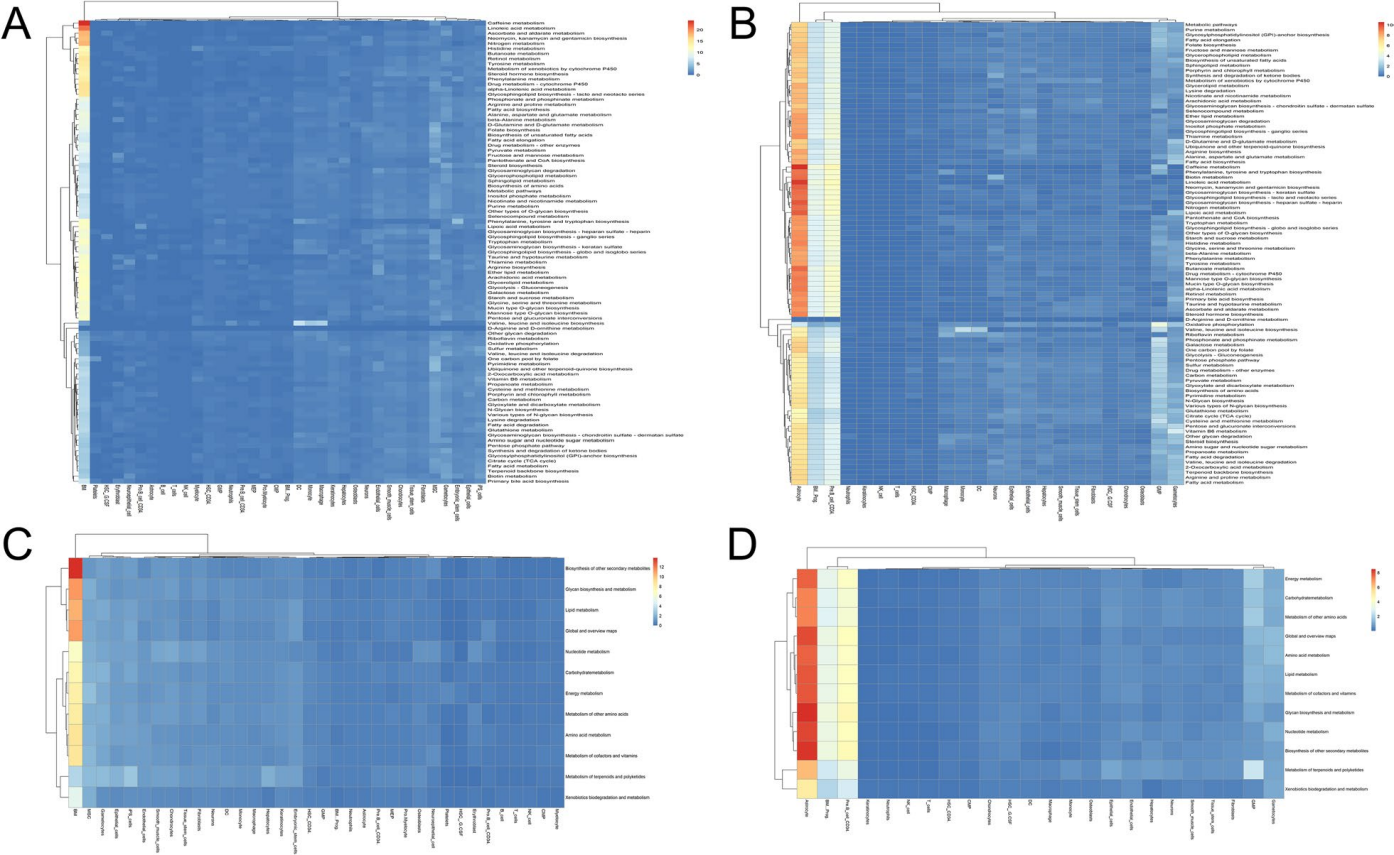
Cell type-specific metabolic reprogramming. **A** Metabolic pathway activities in cell types in the BPH dataset. **B** Metabolic pathway activities in cell types in the healthy control dataset; **C** 11 categories metabolic pathway activities in cell types in the BPH dataset; **D** 11 categories metabolic pathway activities in cell types in healthy control dataset

We conduct to identify the overall features of metabolic pathway variation among the different cell clusters between BPH and control groups. As shown in Fig. 4A, BM cells from BPH patients owned the largest number of metabolic pathways significantly upregulated, including caffeine metabolism, linoleic metabolism (*p* < 0.05, respectively), et al. Meanwhile, as shown in Fig. 4B, astrocyte, BM..PROG and pre.B_cell_CD34 from donors owned the largest number of metabolic pathways significantly upregulated, such as caffeine metabolism and linoleic acid metabolism (*p* < 0.05, respectively). Next, we further characterized the cell cluster-specific metabolic features. According to KEGG classification, above metabolism pathways were divided into 11 categories for comprehensive evaluation. After enrichment analysis, as shown in Fig. 4C, D, BM cells from BPH patients showed higher activity in biosynthesis of other secondary metabolites, glycan biosynthesis and metabolism, and lipid metabolism, while astrocytes from donors showed higher activity in secondary metabolites, glycan biosynthesis and metabolism, and nucleotide metabolism.

### Identification of NRK in human prostate

**Fig. 5:**
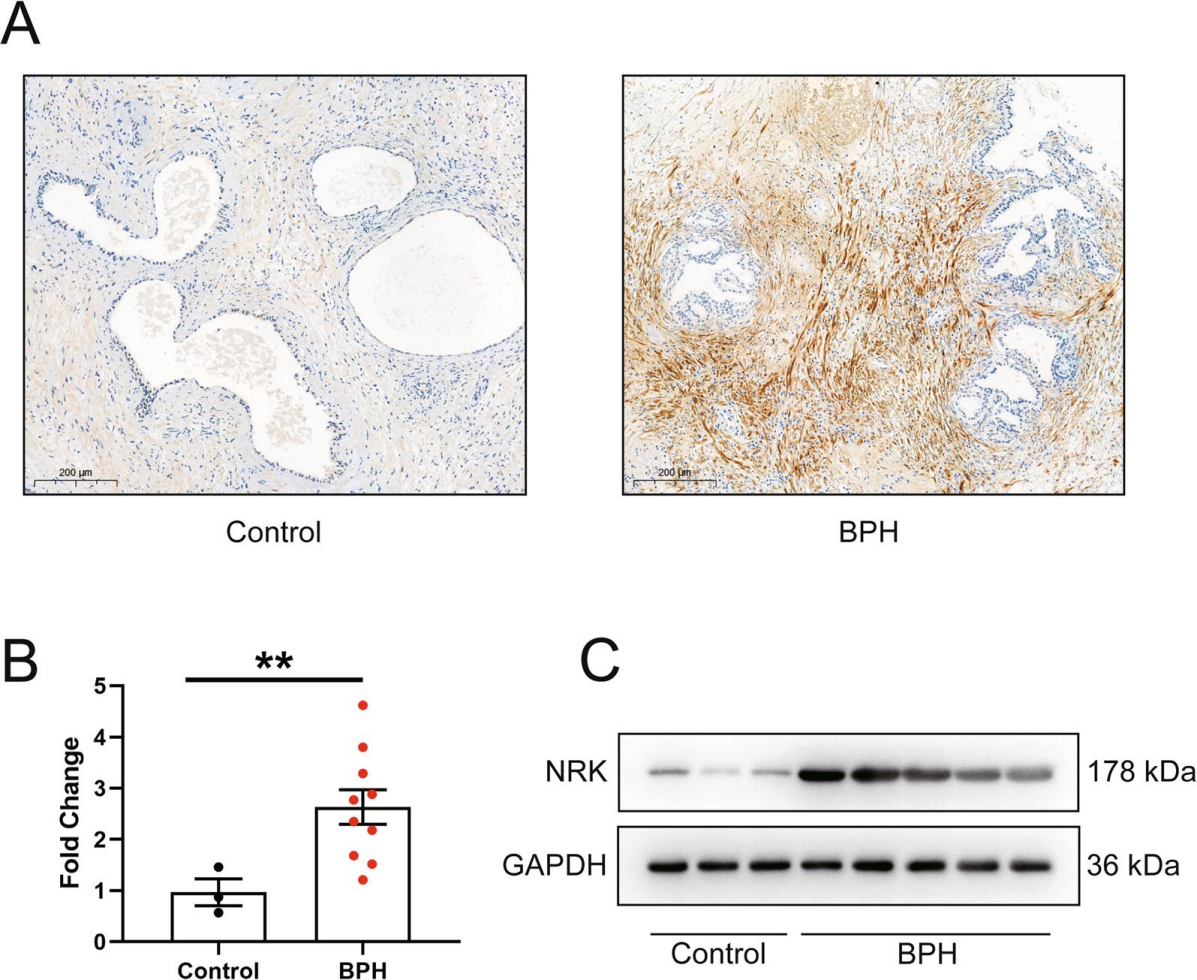
Localization and expression level of NRK in prostate tissue. **A** Typical graph of immunohistochemistry staining of NRK in prostate tissue from BPH patients and donors. The scale bar is 200 μm. **B** The mRNA level of NRK between normal (n = 3) and hyperplastic prostate (n = 10). **Means *p* < 0.01. **C** The typical blotting bands of NRK between normal and hyperplastic prostate. All values are of triplicate measurements and repeated three times with similar results. GAPDH was used as a control

TMA including prostate tissues from 104 BPH/LUTS patients was constructed and IHC staining was conducted. As shown in Fig. 5A, NRK was upregulated in human hyperplastic prostate. Meanwhile, NRK is almost exclusively present in the stroma component and is less expressed in the epithelial component. We further confirm these results in human cell lines. The level of NRK was higher in WPMY-1 cells while less in BPH-1 or RWPE-1 cells (Additional file 4: Figure S3). In addition, RT-PCR and western-blot experiment also demonstrated that NRK was upregulated in human BPH tissues (Fig. 5B, C).

### NRK is associated with clinical parameters in BPH/LUTS patients

We analyzed the correlation between the expression of NRK in TMA (Additional file 4: Figure S4) and clinical parameters of BPH/LUTS patients. As shown in Table 2, the expression level of NRK have a very strong positive correlation with IPSS (*p* = 0.042) while a significant negatively correlation with Q_max_ (*p* = 0.028). However, no significant correlation was shown between NRK and other characters.

**Table 2:** Correlation between expression level of NRK and clinical information of BPH patients

Items	Correlation index	p-value
Age (year)	0.045	0.554
BMI (kg/m^2^)	− 0.172	0.404
Prostatic size (ml)	0.118	0.498
tPSA (ng/ml)	0.135	0.336
fPSA (ng/ml)	0.142	0.267
IPSS	0.248*	0.042
Q_max_ (m/s)	− 0.269*	0.028
PVR (ml)	− 0.125	0.24

### NRK regulates cell proliferation, apoptosis, cycle and migration

**Fig. 6:**
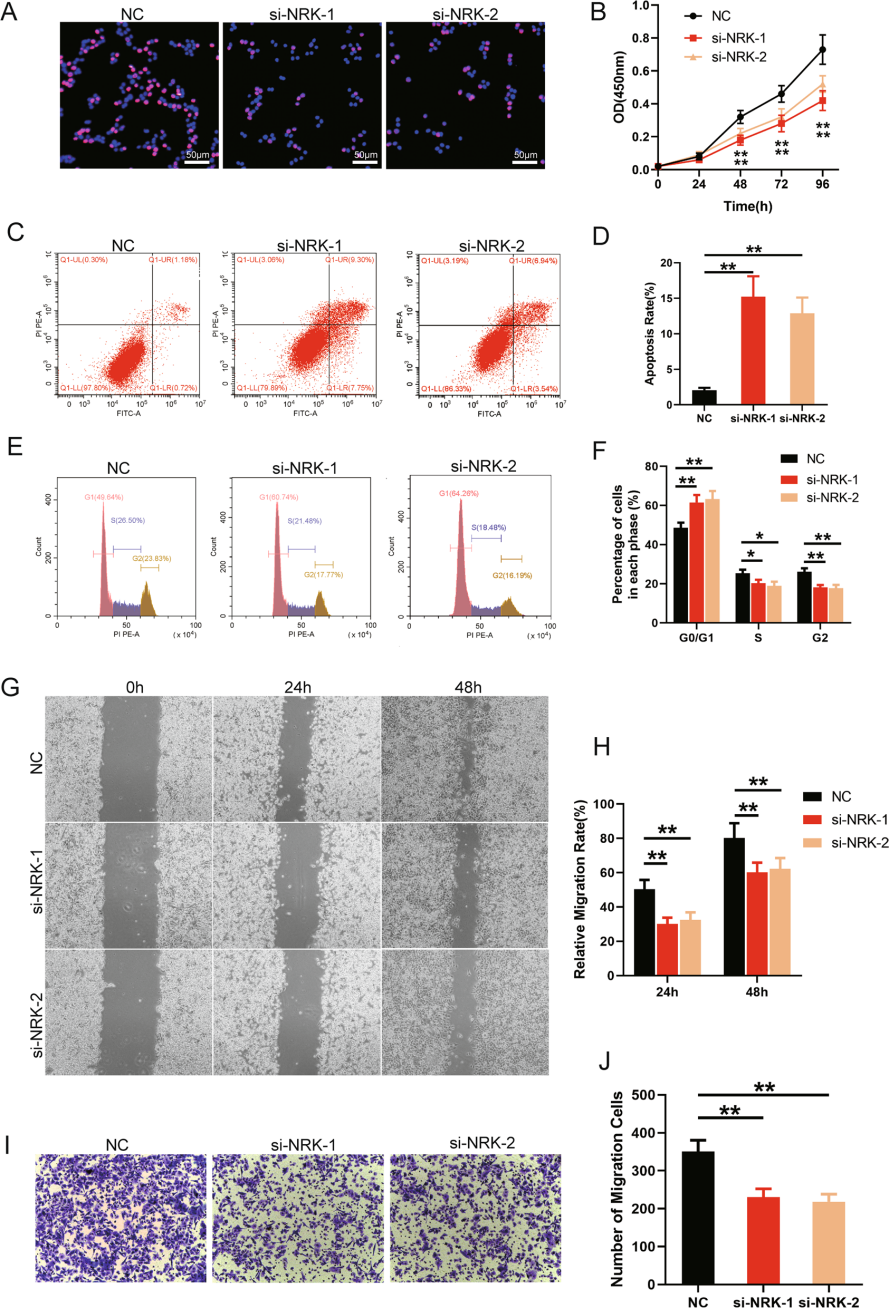
Downregulation of NRK inhibited cell proliferation, induced cell apoptosis, arrest cell cycle and suppressed migration. **A** The typical graph of EdU staining of si-control and si-NRKs groups. Nuclei were stained by DAPI (blue). **B** CCK-8 assay was used to detect the viability of the WPMY-1 cells treated by si-control (black line) and si-NRKs (red line and yellow line). **C** Flow cytometry analysis of alterations of cells apoptosis in WPMY-1 cells transfected with si-control and si-NRKs. PI PE-A in y-axis stands for the fluorescence intensity of propidine iodide (PI) and FITC-A in x-axis stands for the fluorescence intensity of Fluorescein isothiocyanate (FITC) laballed Annexin V. Calculation area of the apoptosis rate was percentage of Annexin V + /PI + cells. **D** Statistical analysis of cell apoptosis rate according to flow cytometry analysis. **E** Flow cytometry analysis for proportion of three cell cycle phase. **F** Statistical analysis of cell cycle stages according to flow cytometry analysis. **G** Wound healing assay of WPMY-1 cells with or without NRK silencing in 24 and 48 h. **H** Statistical analysis of relative migration rate according to wound healing assay. **I** Transwell assay of WPMY-1 cells with or without NRK silencing. **J** Statistical analysis of migration cells according to transwell assay. All values shown are mean ± SD of triplicate measurements and repeated three times with similar results, *Means *p* < 0.05 and **means *p* < 0.01

WPMY-1 cells with NRK knockdown were used to assess the biological functions of NRK. The EdU staining showed that positive cells were decreased in NRK knockdown WPMY-1 cell (Fig. 6A). Meanwhile, CCK-8 assay also confirmed the inhibition of survival after NRK knockdown (Fig. 6B, p < 0.01). Flow cytometry analysis detected that apoptosis rate was higher with NRK knockdown (Fig. 6C, D, p < 0.01). Furthermore, the proportion of G0/G1 phase cells were increased significantly with NRK knockdown (p < 0.01) while the proportion of S phase and G2 phase cells were decreased significantly (p < 0.01 and p < 0.01, respectively) (Fig. 6E, F). In addition, wound healing assay (p < 0.01) and transwell assay (p < 0.01) showed that the migration was attenuated with NRK silencing (Fig. 6G–J).

**Fig. 7:**
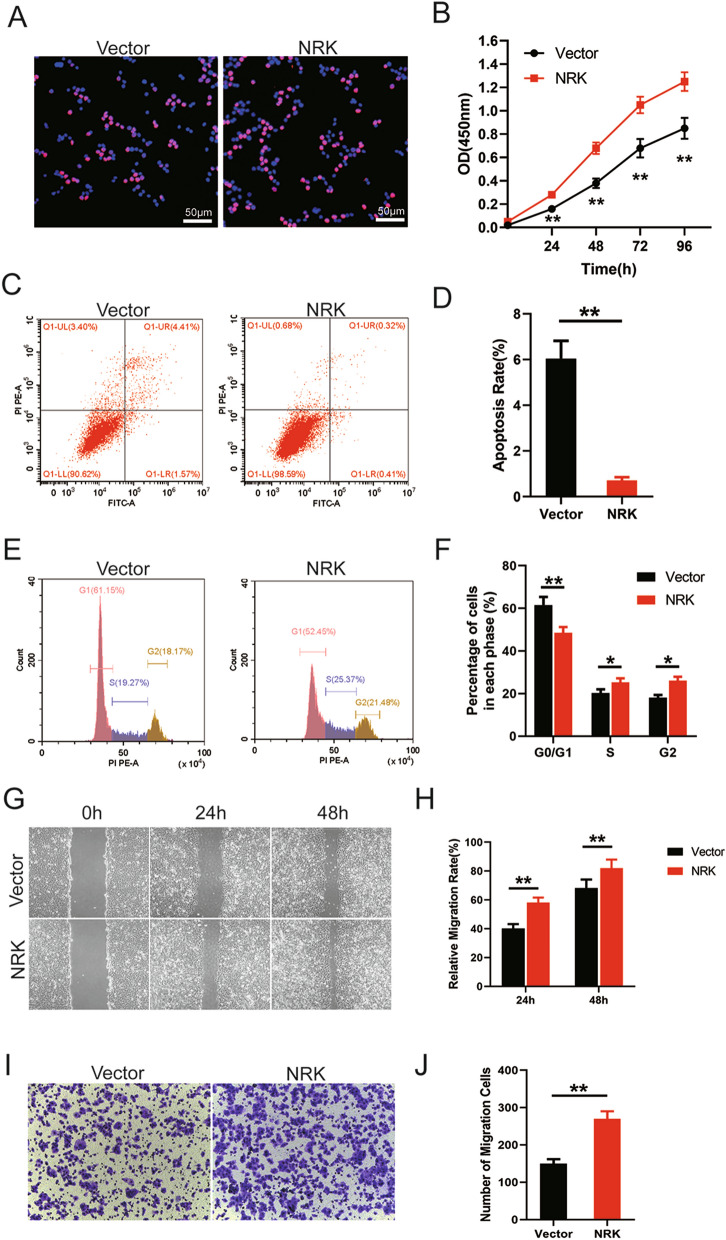
Overexpression of NRK promoted cell proliferation, attenuated cell apoptosis, accelerate cell cycle and increased migration. **A** The typical graph of EdU staining of si-control and si-NRKs groups. Nuclei were stained by DAPI (blue). **B** CCK-8 assay was used to detect the viability of the BPH-1 cells treated by Vector (black line) and NRK-overexpression plasmid (red line). **C** Flow cytometry analysis of alterations of cells apoptosis in BPH-1 cells transfected with Vector and NRK plasmid. PI PE-A in y-axis stands for the fluorescence intensity of propidine iodide (PI) and FITC-A in x-axis stands for the fluorescence intensity of Fluorescein isothiocyanate (FITC) laballed Annexin V. Calculation area of the apoptosis rate was percentage of Annexin V + /PI + cells. **D** Statistical analysis of cell apoptosis rate according to flow cytometry analysis. **E** Flow cytometry analysis for proportion of three cell cycle phase. **F** Statistical analysis of cell cycle stages according to flow cytometry analysis. **G** Wound healing assay of BPH-1 cells with or without NRK overexpression in 24 and 48 h. **H** Statistical analysis of relative migration rate according to wound healing assay. **I** Transwell assay of BPH-1 cells with or without NRK overexpression. **J** Statistical analysis of migration cells according to transwell assay. All values shown are mean ± SD of triplicate measurements and repeated three times with similar results, *means *p* < 0.05 and **means *p* < 0.01

Meanwhile, BPH-1 cells with NRK overexpression were also used to assess the biological functions of NRK. The EdU staining showed that positive cells were increased in NRK-overexpressed BPH-1 cell (Fig. 7A). Meanwhile, CCK-8 assay also confirmed the promotion of survival after NRK overexpression (Fig. 7B, p < 0.01). Flow cytometry analysis detected that apoptosis rate was lesser with NRK overexpression (Fig. 7C, D, p < 0.01). Furthermore, the proportion of G0/G1 phase cells were decreased significantly with NRK overexpression (p < 0.01) while the proportion of S phase and G2 phase cells were increased significantly (p < 0.05 and p < 0.05, respectively) (Fig. 7E, F). In addition, wound healing assay (p < 0.01) and transwell assay (p < 0.01) showed that the migration was promoted with NRK expression (Fig. 7G–J).

**Fig. 8:**
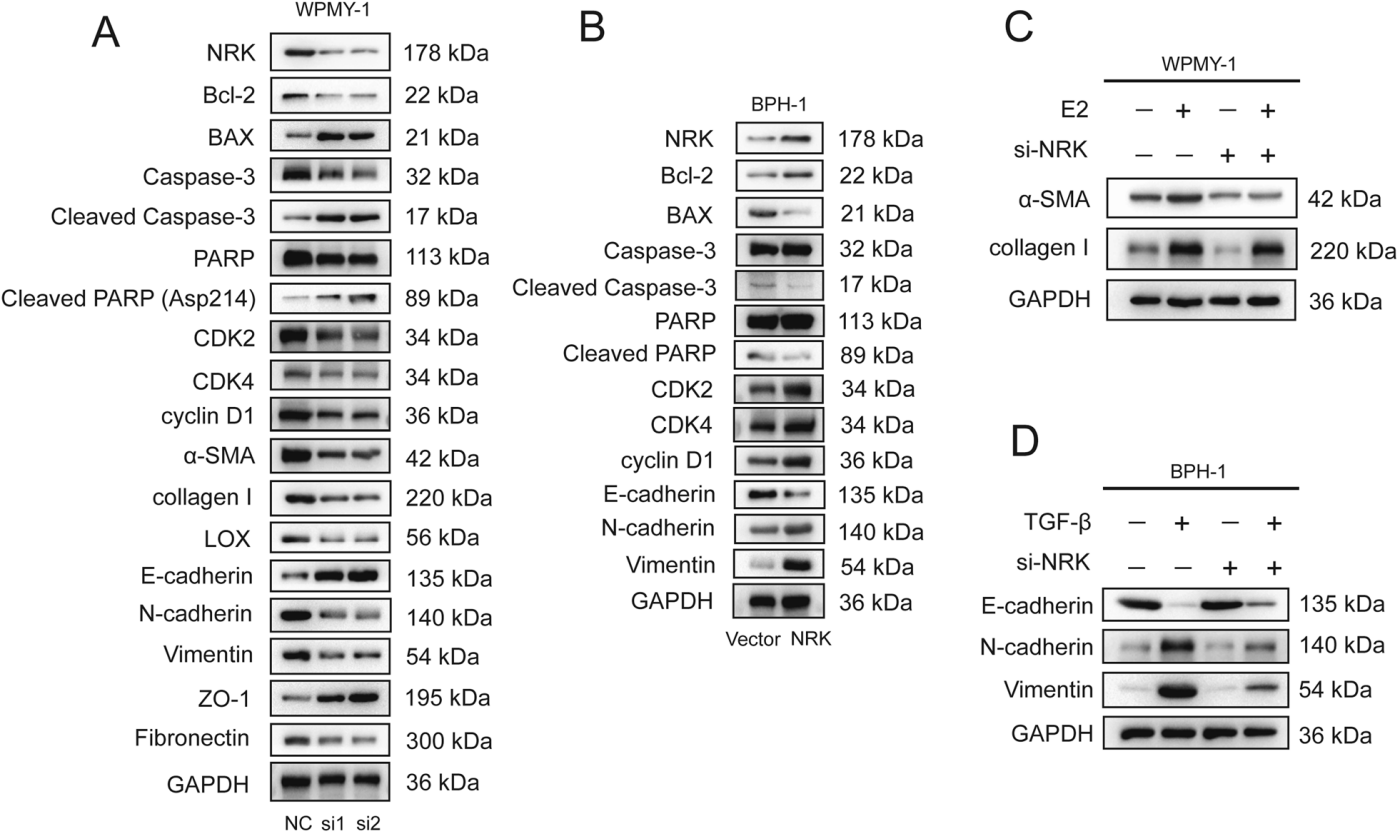
NRK is associated with cell proliferation, apoptosis, cell cycle, fibrosis and EMT process. **A** The typical blotting bands of proteins related to cell proliferation, apoptosis, cell cycle, fibrosis and EMT process among si-control and si-NRKs groups in WPMY-1 cells. **B** The typical blotting bands of proteins related to cell proliferation, apoptosis, cell cycle, and EMT process between vector and NRK plasmid groups in BPH-1 cells. **C** The typical blotting bands of proteins related to fibrosis with or without E2 and si-NRK. **D** The typical blotting bands of proteins related to EMT-process with or without TGF-β and si-NRK. GAPDH was used as a control and all experiments repeated three times with similar results

Then western-blot experiment detected the level of related proteins involved in apoptosis and the cell cycle. Bcl-2, Caspase-3, PARP, CDK2/4 and Cyclin D1 were downregulated while BAX, cleaved Caspase-3, cleaved PARP were upregulated in WPMY-1 cells with NRK knockdown (Fig. 8A). On the other hand, Bcl-2, Caspase-3, PARP, CDK2/4 and Cyclin D1 were upregulated while BAX, cleaved Caspase-3, cleaved PARP were downregulated in BPH-1 cells with NRK overexpression (Fig. 8B).

### NRK contributed to fibrosis and EMT process in prostate cells

With NRK knockdown in WPMY-1 cells, fibrosis related proteins including α-SMA, collagen I and LOX were downregulated (Fig. 8A). In addition, E-cadherin and ZO-1 were upregulated while N-cadherin, Vimentin and Fibronectin were downregulated after NRK knockdown in WPMY-1 cells (Fig. 8A). Meanwhile, E-cadherin was downregulated while N-cadherin and Vimentin were downregulated after NRK overexpression in BPH-1 cells (Fig. 8B).

To further clarify whether NRK plays a role in fibrosis, we used E2 to induce prostate cell fibrosis. The level of α-SMA and collagen I were both increased with E2 administration, while rescued by NRK knockdown (Fig. 8C). Furthermore, BPH-1 cells and TGF-β were used to validated the role of NRK in EMT process. The level of E-cadherin was increased while the level of N-cadherin and Vimentin were decreased after TGF-β induction, however, these alterations could be rescued by NRK knockdown (Fig. 8D).

**Fig. 9:**
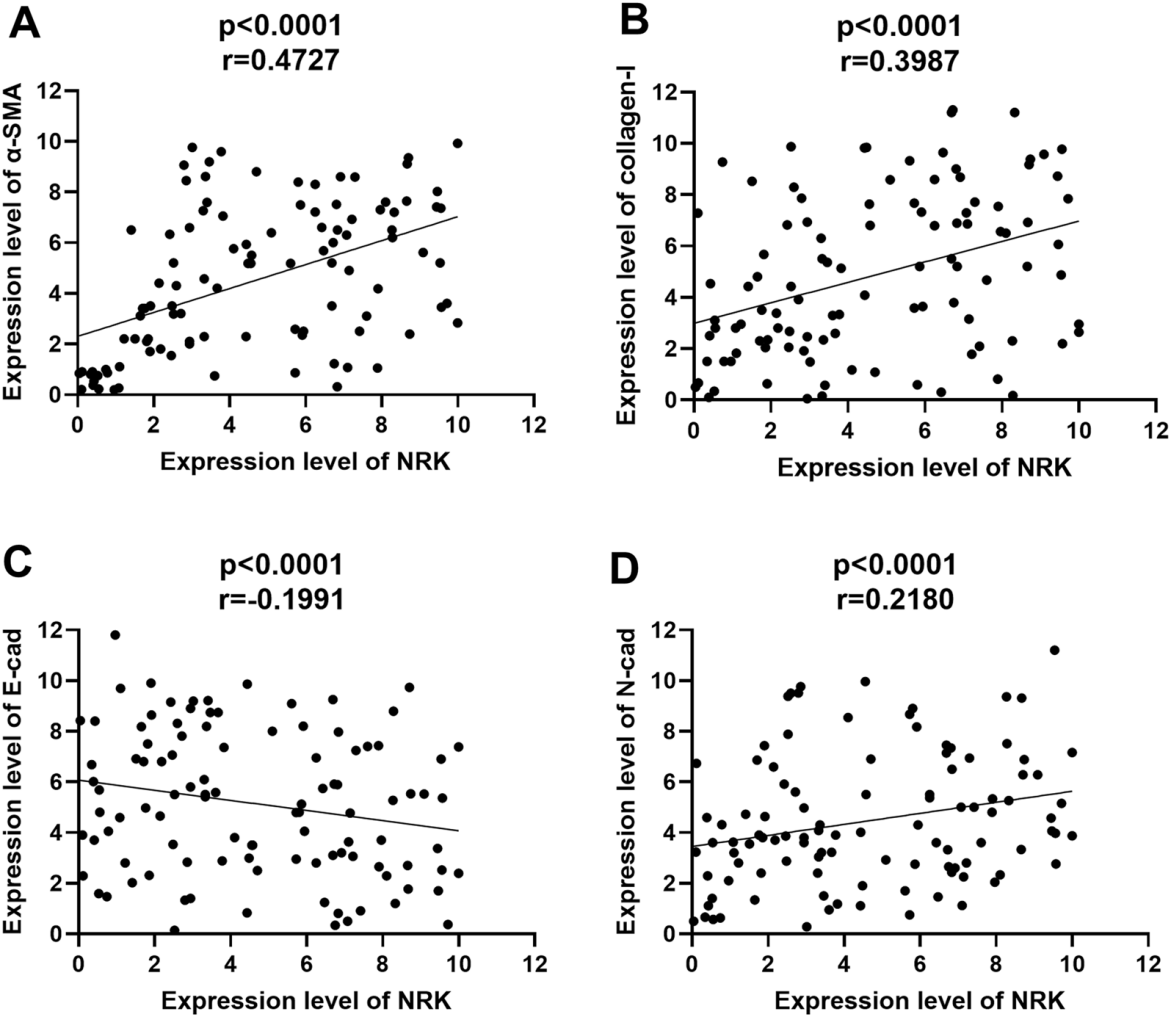
NRK is correlated with fibrosis and EMT process. Correlation analysis between the protein expression level of NRK and fibrosis (or EMT process) related proteins in BPH patients. X axis means the expression level of NRK from 104 BPH patients in TMA. Y axis means the expression level of α-SMA (**A**), collagen-I (**B**), E-cadherin (**C**) and N-cadherin (**D**), respectively

Then, we analyzed the correlation between expression level of NRK and proteins related to fibrosis or EMT process in 104 BPH patients through TMA, respectively (Fig. 9A–D). The correlation coefficient between NRK and α-SMA is 0.4727 (p < 0.0001). The correlation coefficient between NRK and collagen I is 0.3987 (p < 0.0001). The correlation coefficient between NRK and E-cadherin is − 0.1991 (p = 0.0327). The correlation coefficient between NRK and N-cadherin is 0.2180 (p < 0.0001).

## Discussion

In the current study, we are the first time to identify the expression and functional activity of NRK in human hyperplastic prostate tissues. We conducted bioinformatic analysis using bulk RNA-seq and single cell RNA-seq data to compare the DEGs between hyperplasia and normal prostate. A total of 17 DEGs were identified among three datasets. Meanwhile, we performed single cell RNA-seq analysis and revealed the communication between cells clusters and cell type-specific metabolic pathway. The expression level of DEGs were analysis in three cell clusters separately (epithelial cells, fibroblasts and SM cells) with NRK remarkably upregulated in fibroblasts and SM cells of hyperplasia prostate. The expression, localization and functional activity of NRK were further explored using TMA and cultured human prostate cell lines. Our novel data demonstrated that the upregulation of NRK in the enlarged prostate could contribute to the development of BPH via prompting cell proliferation, cell cycle, migration, fibrosis and EMT process, instead of inhibiting cell apoptosis.

Although aging and testosterone are most significantly risk factors, the underlying mechanism of BPH/LUTS is remain undefined. In recent years, with the development of bioinformatic analysis for many diseases, many studies exploring the pathophysiology or potential biomarkers of BPH/LUTS through analyzing the DEGs between human hyperplastic and normal prostate [14, 18]. However, study using single cell RNA data or combining multiple platforms or datasets is few. In current study, we merged the DEGs from three bulk RNA-seq datasets and focused on 17 genes, which made data more comprehensive and precise. Then scRNA-seq dataset, GSE172357, was used for further analysis, which was published recently. Joseph and their colleagues analyzed this dataset and demonstrated some novel fibroblasts with specialized distribution and microenvironment interactions, which may give some cues for exploring the development of BPH/LUTS [20]. Our current data confirmed their results and give some new findings, such as communication and difference of proportion among cell clusters. Since interactions among cell clusters play important roles in BPH development, our novel findings may provide foundation for further research. Meanwhile, more evidences demonstrated that metabolic disturbances were significantly associated with BPH development. According to the methods and rationales developed by Xiao and colleagues [38], we standardized and integrated the expression levels of metabolic genes in each metabolic pathway, through which the metabolic activity score is obtained to reflect the metabolic flux of each metabolic pathway. These methods could reveal the heterogeneity of metabolic features among different cell clusters under many disease conditions [38, 39], while it was less studied in BPH. In current study, we screen the cell type-specific metabolic pathway and get some novel findings. Bone marrow (BM) from BPH patients showed greatly changes in metabolic pathways. BM contribute a significant proportion of cells in the skin [40]. The normal skin has long been known to contain BM-derived cells that are involved in host defense and inflammatory processes, including wound healing. However, recent study also demonstrated that BM act as noninflammatory cells in skin, in which they mainly exist as fibroblast-like form in dermis and as keratinocyte phenotype in epidermis [41]. However, BM cells is less studied in hyperplastic prostate, which was worthy of further explorations.

Since SM, fibroblasts and epithelial cells are most predominantly in human prostate tissues, we explored the above 17 DEGs in these three cell clusters. As expected, the proportion of SM and fibroblasts are significantly higher in hyperplastic prostate (*p* < 0.05, respectively), which further confirmed the changes in stromal component during hyperplasia. Although these 17 DEGs were up- or downregulated in hyperplastic prostate, the changes in single cell cluster were not completely same with that in whole tissues, this suggested that combined analysis could give more precise inspiration for further study. Then we focused the genes whose changes were same between single cell cluster and tissue including BMP5, NELL2, NRK, LUM, TBX4, PDK4 and MSMO1. BMP5, bone morphogenetic protein 5, was found upregulated in hyperplastic prostate tissue from three datasets (10.3-, 12.6- or 4.3-fold for GSE7307, GSE119195 and GSE132714, respectively). A previous study demonstrated that BMP5 was upregulated in hyperplastic prostate tissue and modulated cell proliferation and the EMT process through the BMP/Smad signaling pathway [42]. NELL2, neural epidermal growth factor-like like 2, was upregulated in hyperplastic prostate tissue from three datasets (7.5-, 12.3- or 3.8-fold for GSE7307, GSE119195 and GSE132714, respectively). A previous study also confirmed NELL was upregulated in BPH and enhanced cell proliferation and inhibited a mitochondria-dependent cell apoptosis via the ERK pathway [15]. Both BMP5 and NELL2 were also identified by other studies and played important roles in development of BPH. LUM, lumican, was upregulated in hyperplastic prostate tissue from three datasets (2.6-, 2.5- or 2.2-fold for GSE7307, GSE119195 and GSE132714, respectively). LUM was presented in the reactive stroma surrounding prostate primary tumors and played a restrictive role on cancer progression [43]. In addition, LUM also enhanced the proliferation, migration and differentiation of synovial fibroblasts [44]. Recent studies confirmed that LUM was abundantly expressed in stroma of hyperplastic prostate [45], however, the role of LUM in BPH are remain undefined. TBX4, T-box transcription factor 4, encodes a member of the evolutionarily conserved family of T-box–containing transcription factors, was upregulated in hyperplastic prostate tissue from three datasets (2.5-, 2.2- or 2.2-fold for GSE7307, GSE119195 and GSE132714, respectively). The most investigations of TBX4 focused on diseases of bone development and respiratory system [46], however, its role in BPH was not yet elucidated. PDK4, pyruvate dehydrogenase kinase 4, is one of the isozymes of pyruvate kinase and has been widely investigated in various disease. Recent studies found that downregulation of PDK4 induced by high glucose promotes prostate cell proliferation and EMT process, which may play important roles in BPH development [47]. Consistently, our current study also found that PDK4 was downregulated in hyperplastic prostate tissue from three datasets (0.2-, 0.2- or 0.1-fold for GSE7307, GSE119195 and GSE132714, respectively). MSMO1, methylsterol monooxygenase 1, was downregulated in hyperplastic prostate tissue from three datasets (0.4-, 0.3- or 0.2-fold for GSE7307, GSE119195 and GSE132714, respectively). MSMO1 was a synonym for sterol-C4-methyl oxidase analog (SC4MOL), plays important role in the normal synthesis of cholesterol [48]. Recent studies demonstrated that MSMO1 has participated the development of various cancer [49–52], however, its role in prostate is not reported. Then we focused on NRK, which was a markedly upregulated DEGs from analysis of scRNA-seq and bulk RNA-seq (3.9-, 7.1- or 4.1-fold for GSE7307, GSE119195 and GSE132714, respectively). We have searched for literatures and found that NRK was studied more in placenta, while none study investigated NRK in prostate, which draw our interesting. Indeed, we confirmed that NRK was upregulated in human hyperplastic prostate compared with normal donors. The study about the NRK was few, including in the prostate disease. Our novel data also demonstrated that NRK was almost localized in stromal component of prostate and confirmed its upregulation in stromal component (including SM cells and fibroblasts).

Considering the localization of NRK in hyperplastic tissues, we used WPMY-1 cell lines to explore the potential function of NRK in stromal cells. The present study created a NRK deficiency cell model using transfection with siRNA to knockdown NRK. The silencing of NRK inhibited WPMY-1 proliferation drastically and suffered a significant higher rate of apoptosis. Meanwhile, remarkable cell cycle arrest at G0/G1 phase was observed when NRK silencing. Consistently, overexpression of NRK in BPH-1 cells showed opposite results. Furthermore, our novel western-blotting data also confirmed these changes in proliferation, apoptosis (Bcl-2, BAX, caspase-3 and PARP) and cell cycle (CDK2, CDK4 and cyclin D1) by detecting their protein expression levels. In past, initial studies focused the function of NRK on placenta proliferation and demonstrated its pro-proliferation role in NRK knockout model, which was on the contrary to out novel data [22, 24–26]. However, further studies also demonstrated its different expression pattern in multiple organs and multiple roles in various disease, which may suggest its distinct functions among different diseases.

Since tissue fibrosis and EMT process is strictly associated with stromal component in which NRK is mostly localized, we further investigated whether NRK participates in tissue fibrosis or EMT process. Interestingly, our novel data found the markers of fibrosis and EMT were downregulated in human prostate stromal cells when NRK silencing, while markers of EMT were upregulated in prostate epithelial cells with NRK overexpression. Consistently, the migration of prostatic cells was enhanced by NRK overexpression while attenuated by NRK knockdown. In order to confirm the role of NRK in tissue fibrosis and EMT, we induced fibrosis in human prostate stromal cell lines using E2, with or without NRK silencing. As previous described [36], fibrosis markers (α-SMA and collagen I) were upregulated with E2 administration, while knockdown of NRK rescued these changes. Similarly, TGF-β, a frequently used inducer for EMT process [35], have successfully promoted EMT process in prostate epithelial cells, which was reflected by expression changes in E-cadherin, N-cadherin and Vimentin. However, changes of protein level in these markers were rescued with by knockdown of NRK. These novel findings suggested that NRK play important roles in tissue fibrosis and EMT process, and then participates in BPH progress. Similar findings about promotion of fibrosis and EMT process were demonstrated in previous studies, such as NELL2 and BMP5 [15, 42]. However, the exact mechanism of fibrosis and EMT process is remain undefined. Therefore, further study about the mechanism that NRK participating in prostate fibrosis and EMT process are needed. Currently, the first-line therapies for BPH are based on the physiology of the prostate with α1-blockers (reduce the prostate tone) and 5α-reductase inhibitors (reduce the prostate volume). However, these treatments cannot completely relief tissue fibrosis or even prevent the progression of BPH. The present study identified NRK upregulated in hyperplastic prostate tissue and played important roles in prostate fibrosis and EMT process. Therefore, treatment based on NRK could play a more important role for the future therapy for BPH during routine clinical practice.

Interestingly, our novel data demonstrated that the expression level of NRK was significantly correlated with IPSS and Q_max_, whereas there was no significant correlation between other clinical characteristics and NRK expression. Further investigation showed that NRK expression level was negatively correlated with expression level of E-cad, while positively correlate with α-SMA, collagen I and N-cad in BPH tissues, indicating a significantly positive correlation between NRK and prostate fibrosis, as well as EMT process. Meanwhile, these results were consistent with above findings. These correlations among NRK and clinical characters indicated its potential roles in multiple biological processes of BPH/LUTS, which was worthy of further study.

There are several factors and limitations that may have influenced the results of the current study. First, three bulk RNA-seq dataset were used for analysis. Detection platforms and probes are different among datasets, which may cause some bias in our study. Second, the characters of patients are distinct. For example, hyperplastic prostate might have cancer-related genetic changes since they were obtained from patients undergoing cystoprostatectomy for infiltrating bladder cancer. Meanwhile, some control prostate tissue from these datasets are donors while others are age-matched patients. Therefore, more homogeneous datasets are need for further study. Third, BPH animal models that stimulating human BPH progress could be used in further study to explore the expression and function of NRK in prostate comprehensively. Meanwhile, manipulating gene expression in animal prostate could be better understanding the role of NRK in development of BPH/LUTS. Fourth, most hyperplastic tissues are successfully preserved and ultimately utilized for further study. However, since BPH samples were collected during TURP operation, some tissues were not used for further because their poor quality which was investigated by pathologist or us. Finally, the downstream genotype effects and rescue experiments would deeply reveal the underlying mechanisms and related signaling pathways that NRK participated in BPH/LUTS. We believe that it is very interesting and meaningful to explore in the future.

Collectively, this is the first study to reveal both NRK gene and protein expression increased in hyperplastic prostate, while knockdown of NRK inhibited prostatic stromal cell proliferation and cycle, promoted cell apoptosis. Meanwhile, NRK play important roles in prostate tissue fibrosis and EMT process, which may participate in the development of BPH and it could be rediscovered as new therapeutic targets for BPH.

### Supplementary Information

**Additional file 1: Table S1.** List of primary antibodies.

**Additional file 2: Table S2.** List of siRNA sequences.

**Additional file 3: Table S3.** Primer sequences used to amplify target genes in human by PCR.

**Additional file 4: Figure S1.** The mRNA level of NRK in WPMY-1 cells with or without siNRK. **Figure S2.** The mRNA level of NRK in BPH-1 cells with or without NRK overexpression. **Figure S3.** The expression level of NRK in prostatic stromal and epithelial cells. **Figure S4.** The IHC staining of NRK in TMA. The scale bar is 2 mm. **Figure S5.** Correlation analysis between the protein expression level of NRK and clinical characters of BPH patients.

## Data Availability

The data used to support the findings of this study are available from the corresponding author upon request.

## Abbreviations

BPH: Benign prostatic hyperplasia
LUTS: Lower urinary tract symptoms
α1-blockers: α1-Adrenoceptor antagonists
5-ARIs: 5α-Reductase inhibitors
ECM: Extracellular matrix
EMT: Epithelial-mesenchymal transition
NRK: Nik related kinase
GCK: Germinal center kinase
DMSCs: Dermal mesenchymal stem cells
PBMCs: Peripheral blood-derived mononuclear cells
MMPs: Matrix metalloproteinases
TNBC: Triple-negative breast cancer
GEO: Gene Expression Omnibus
DEGs: Differentially expressed genes
GO: Gene Ontology
KEGG: Kyoto Encyclopedia of Genes and Genomes
tSNE: T-distributed stochastic neighbor embedding
FBS: Fetal bovine serum
siRNAs: Small interfering RNAs
PMSF: Phenylmethanesulphonylfluoride
SDS-PAGE: Sodium dodecyl sulfate–polyacrylamide
PVDF: Polyvinylidene fluoride
ECL: Enhanced chemiluminescence
Bio-Rad: Bio-Rad Molecular Imager® ChemiDoc™ XRS + System and Quantity One® SW 1-D Analysis Software
IOD: Integrated optical density
BP: Biological process
CC: Cellular component
MF: Molecular function
PV: Prostate volume
fPSA: Free prostate-specific antigen
