# Supplementary material for: Identification and functional activity of Nik related kinase (NRK) in benign hyperplastic prostate
Source: J Transl Med. 2024 Mar 9;22:255. doi: 10.1186/s12967-024-05048-3 (PMC11367987; doi:10.1186/s12967-024-05048-3)
Supplement: Supplementary file 1 — Additional file 1: Table S1. List of primary antibodies. [file 12967_2024_5048_MOESM1_ESM.docx]

| Table S1. List of primary antibodies. | | | | |
| --- | --- | --- | --- | --- |
| Protein target | Name of antibody | Manufacturer and catalog | Species raised in; monoclonal or polyclonal | Dilution used |
| NRK | NRK antibody | Affinity  #DF2670 | Rabbit  Polyclonal | 1:1000 (WB)  1:100 (IHC) |
| Bcl-2 | Bcl-2 antibody | ABclonal A21592 | Rabbit  Polyclonal | 1:1000 (WB) |
| BAX | BAX antibody | ABclonal A0207 | Rabbit  Polyclonal | 1:1000 (WB) |
| Casepase-3 | Anti-pro Caspase-3 antibody | Abcam ab32150 | Rabbit  Monoclonal | 1:1000 (WB) |
| Cleaved Casepase-3 | Anti-Caspase-3 antibody | Abcam ab32351 | Rabbit  Monoclonal | 1:1000 (WB) |
| PARP | Anti-PARP1  antibody | Abcam ab227244 | Rabbit  Polyclonal | 1:1000 (WB) |
| Cleaved PARP | Cleaved PARP (Asp214) antibody | ABclonal A22535 | Rabbit  Polyclonal | 1:1000 (WB) |
| CDK2 | CDK2 antibody | ABclonal A0094 | Rabbit  Monoclonal | 1:1000 (WB) |
| CDK4 | CDK4 antibody | ABclonal A23521 | Rabbit  Monoclonal | 1:1000 (WB) |
| Cyclin D1 | Cyclin D1 antibody | ABclonal A22104 | Rabbit  Monoclonal | 1:1000 (WB) |
| α-SMA | α-Smooth Muscle Actin antibody | ABclonal A17910 | Rabbit  Monoclonal | 1:1000 (WB)  1:100 (IHC) |
| Collagen-I | Collagen I/COL1A2 antibody | ABclonal A21059 | Rabbit  Monoclonal | 1:1000 (WB)  1:100 (IHC) |
| LOX | LOX antibody | ABclonal A11504 | Rabbit  Monoclonal | 1:1000 (WB) |
| E-cadherin | E-cadherin antibody | ABclonal A22850 | Rabbit  Monoclonal | 1:1000 (WB)  1:100 (IHC) |
| N-cadherin | N-cadherin antibody | ABclonal A19083 | Rabbit  Monoclonal | 1:1000 (WB)  1:100 (IHC) |
| Vimentin | Vimentin antibody | ABclonal A19607 | Rabbit  Monoclonal | 1:1000 (WB) |
| ZO-1 | ZO-1 antibody | ABclonal A25202 | Rabbit  Polyclonal | 1:1000 (WB) |
| Fibronectin | Fibronectin antibody | ABclonal A12977 | Rabbit  Monoclonal | 1:1000 (WB) |
| GAPDH | GAPDH antibody | ABclonal A19056 | Rabbit  Monoclonal | 1:2000 (WB) |
