# Supplementary material for: Identification and functional activity of Nik related kinase (NRK) in benign hyperplastic prostate
Source: J Transl Med. 2024 Mar 9;22:255. doi: 10.1186/s12967-024-05048-3 (PMC11367987; doi:10.1186/s12967-024-05048-3)
Supplement: Supplementary file 2 — Additional file 2: Table S2. List of siRNA sequences. [file 12967_2024_5048_MOESM2_ESM.docx]

Table S2. List of siRNA sequences.

| Symbol | Sense sequences | Antisense sequences |
| --- | --- | --- |
| Si-NRK-1 | 5’-GGACCAAGAACUUCAACAATT-3’ | 5’-UUGUUGAAGUUCUUGGUCCTT-3’ |
| Si-NRK-2 | 5’-GGCUAAUGAUGUUUGUAAATT-3’ | 5’-UUUACAAACAUCAUUAGCCTT-3’ |
| Si-NRK-3 | 5’-GGAUGGUGAUUAUGUUGAATT-3’ | 5’-UUCAACAUAAUCACCAUCCTT-3’ |
| Si-NRK-4 | 5’-GAAUAUUCUCACUAGAUAATT-3’ | 5’-UUAUCUAGUGAGAAUAUUCTT-3’ |
| Control siRNA (si-con) | 5’-UUCUCCGAACGUGUCAGGUTT-3’ | 5’-ACGUGACACGUUCGGAGAATT-3’ |
