# Supplementary material for: Identification and functional activity of Nik related kinase (NRK) in benign hyperplastic prostate
Source: J Transl Med. 2024 Mar 9;22:255. doi: 10.1186/s12967-024-05048-3 (PMC11367987; doi:10.1186/s12967-024-05048-3)
Supplement: Supplementary file 3 — Additional file 3: Table S3. Primer sequences used to amplify target genes in human by PCR. [file 12967_2024_5048_MOESM3_ESM.docx]

| Table S3. Primer sequences used to amplify target genes in human by PCR. | |
| --- | --- |
| Target Gene | Primer Sequence |
| NRK |  |
| forward | 5’-TAGTGGATTTTGGAGTGAGTGC-3’ |
| reverse | 5’-CTTCTGTAATCATAGGAACACC-3’ |
| GAPDH |  |
| forward | 5’-TGTGTCCGTCGTGGATCTGA-3’ |
| reverse | 5’-TTGCTGTTGAAGTCGCAGGAG-3’ |
