# Supplementary material for: Identification and functional activity of Nik related kinase (NRK) in benign hyperplastic prostate
Source: J Transl Med. 2024 Mar 9;22:255. doi: 10.1186/s12967-024-05048-3 (PMC11367987; doi:10.1186/s12967-024-05048-3)
Supplement: Supplementary file 4 — Additional file 4: Figure S1. The mRNA level of NRK in WPMY-1 cells with or without siNRK. Figure S2. The mRNA level of NRK in BPH-1 cells with or without NRK overexpression. Figure S3. The expression level of NRK in prostatic stromal and epithelial cells. Figure S4. The IHC staining of NRK in TMA. The scale bar is 2 mm. Figure S5. Correlation analysis between the protein expression level of NRK and clinical characters of BPH patients. [file 12967_2024_5048_MOESM4_ESM.docx]

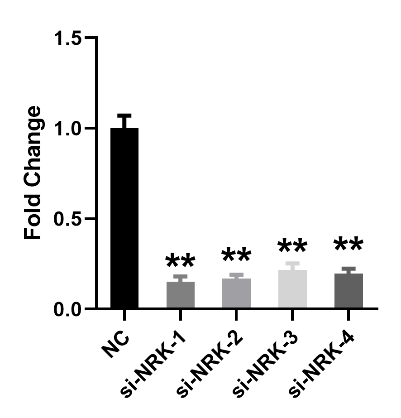


Figure S1. The mRNA level of NRK in WPMY-1 cells with or without siNRK. ** means p value < 0.01 compared with NC group. All values are of triplicate measurements and repeated three times with similar results.


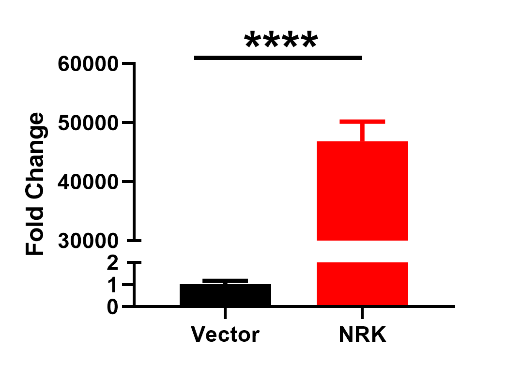


Figure S2. The mRNA level of NRK in BPH-1 cells with or without NRK overexpression. **** means p value < 0.0001 compared with Vector group. All values are of triplicate measurements and repeated three times with similar results.


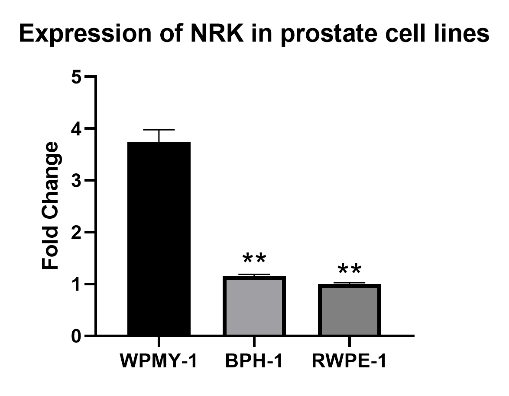


Figure S3. The expression level of NRK in prostatic stromal and epithelial cells.

** means p value < 0.01 compared with WPMY-1 group. All values are of triplicate measurements and repeated three times with similar results.


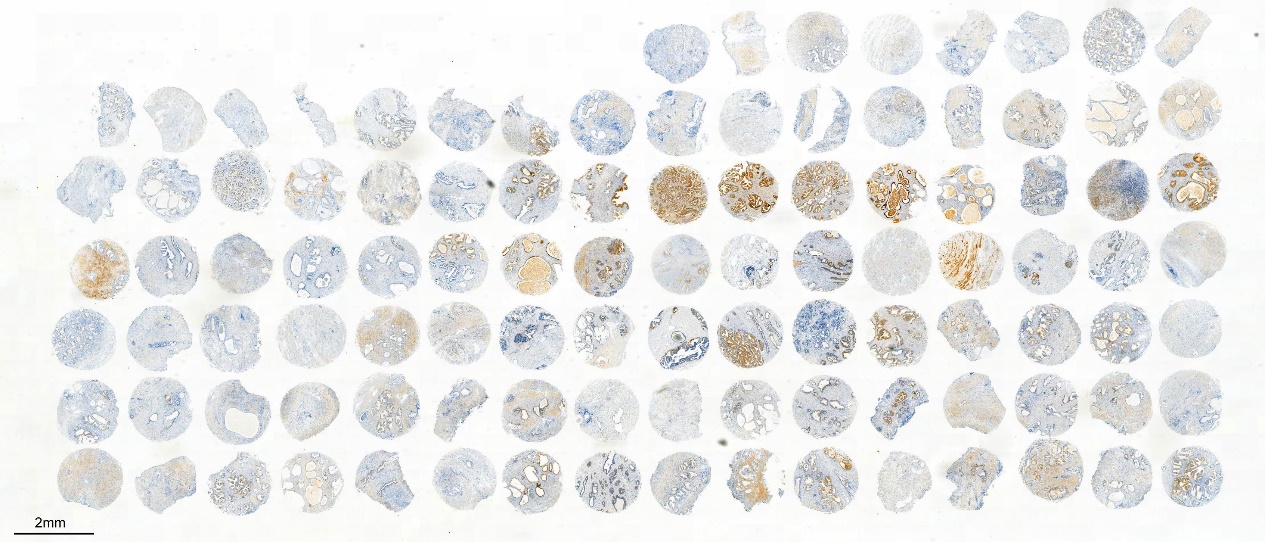


Figure S4. The IHC staining of NRK in TMA. The scale bar is 2 mm.


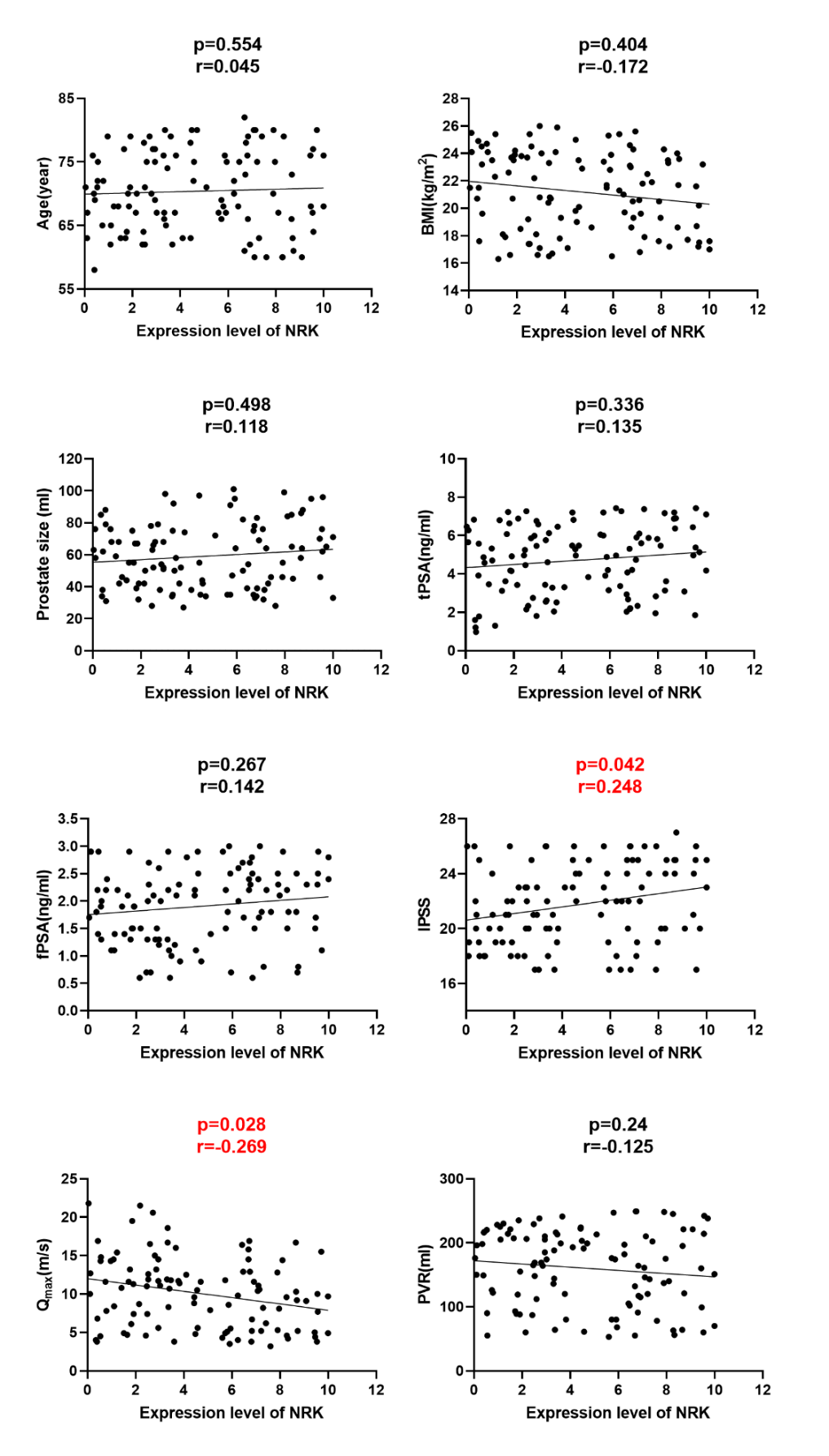


Figure S5. Correlation analysis between the protein expression level of NRK and clinical characters of BPH patients. X axis means the expression level of NRK from 104 BPH patients in TMA. Y axis means the age, BMI, prostate size, tPSA, fPSA, IPSS, Q_max_ and PVR, respectively.
